# Beneficial effects of N-acetylcysteine on protease-antiprotease balance in attenuating bleomycin-induced pulmonary fibrosis in rats

**DOI:** 10.22038/IJBMS.2020.39031.9261

**Published:** 2020-03

**Authors:** Ritu Kulshrestha, Apoorva Pandey, Amteshwar Jaggi, Surendra Bansal

**Affiliations:** 1 Department of Pathology, V. P. Chest Institute, University of Delhi, Delhi-110007, India; 2 Department of Pharmaceutical Sciences and Drug Research, Punjabi University, Patiala, Punjab, India; 3 Department of Biochemistry, V. P. Chest Institute, University of Delhi, Delhi-110007, India

**Keywords:** Bleomycin, MMP-9, N-acetylcysteine, Pulmonary fibrosis, TGF-β1, TIMPs

## Abstract

**Objective(s):**

The role of N-acetylcysteine (NAC) as an anti-oxidant in attenuating bleomycin-induced pulmonary fibrosis has been reported. However, its effect on parenchymal remodeling via regulating the protease-antiprotease balance is not fully defined. Therefore, the present study was designed to explore the possible role of matrix metalloproteinases (MMP), tissue inhibitors of metalloproteinases (TIMP) and transforming growth factor-β1 (TGF-β1) pathway and their modulation by NAC in attenuating bleomycin-induced pulmonary fibrosis in rats.

**Materials and Methods:**

Bleomycin sulphate (7 units/kg) was instilled inside the trachea to induce pulmonary fibrosis. The time course of TGF-β1, MMP-9, TIMP-1,3 mRNA and protein expression, TGF-β1 and hydroxyproline levels were evaluated on days 7, 14, and 28. NAC (0.3 mmol/kg and 3 mmol/kg) was administered in bleomycin-instilled animals.

**Results:**

NAC treatment significantly attenuated bleomycin-induced histopathological changes by decreasing interstitial inflammation and reducing the deposition of extracellular matrix proteins such as collagen. Moreover, it increased the mRNA and protein expression of MMP-9 and decreased the expression of TIMP-1,3 in alveolar epithelial cells (AECs), interstitial macrophages and inflammatory cells. Indeed, there was decrease in the MMP-9/TIMP ratio in bleomycin-instilled rats, which increased with NAC treatment. Moreover, NAC attenuated bleomycin-induced increased expression of TGF-β1 and total lung collagen levels.

**Conclusion:**

NAC attenuates bleomycin-induced pulmonary fibrosis by normalizing the protease-antiprotease balance and favoring the degradation of collegen to reduce fibrosis.

## Introduction

N-acetylcysteine (NAC), a glutathione precursor, is an antioxidant and it is clinically employed in paracetamol toxicity, cystic fibrosis and chronic obstructive pulmonary diseases. A number of preclinical studies have shown the expanding therapeutic potential of NAC (1, 2). Moreover, its usefulness in attenuating bleomycin-induced pulmonary fibrosis has also been documented (3, 4). Nevertheless, the precise mechanisms involved in inducing its beneficial effects in bleomycin-induced pulmonary fibrosis are not precisely known. 

Pulmonary fibrosis is a heterogenous group of lung disorders characterized by chronic inflammation, epithelial mesenchymal transition, aberrant remodeling and irreversible destruction of the lung architecture that ultimately leads to disruption of gas exchange and death from respiratory failure (5). Pathogenesis of pulmonary fibrosis is ill-defined and is linked to abnormal fibroblast response mechanisms. Matrix metalloproteinases (MMPs)-2, 7, 8, and 9, are increasingly implicated in lung tissues that are inflamed and/or undergoing repair and remodeling (6). In addition to direct proteolysis of the extracellular matrix (ECM), these MMPs play a significant role in leukocyte activation, chemokine processing and cell migration during the immunopathogenesis and repair processes (7, 8). The tissue inhibitors of metalloproteinases (TIMPs) naturally inhibit the MMPs, and favor local ECM accumulation by playing a role in cell proliferation, migration, invasion, and apoptosis. Of the four TIMPs, TIMP-3 has the broadest inhibition spectrum (9). Resolution of inflammation is associated with enhanced TIMP expression; however, restoration of normal tissue architecture does not always necessarily follow. In idiopathic pulmonary fibrosis (IPF) tissues, increased TIMP-3 expression has been observed in fibroblastic foci and ECM (10). Dysregulation of the proteolytic-antiproteolytic system is observed in asthma, chronic obstructive pulmonary disease, pulmonary fibrosis and lung cancer (11-13). The role of protease-antiprotease balance at different phases of lung injury and repair during the development of lung fibrosis remains to be elaborated.

Transforming growth factor-β1 (TGF-β1) signaling is an important inducer of fibroblast proliferation, epithelial myoepithelial transition (EMT) (14, 15) and ECM accumulation (16, 17)the pulmonary content of mRNAs encoding fibronectin, alpha 2I procollagen and alpha 1III procollagen was increased. The increases were greater and occurred earlier in C57Bl/6 mice compared to BALB/c mice. Fibronectin mRNA increased 12-fold in C57Bl/6 mice and only 3-fold in BALB/c mice, whereas alpha 1III procollagen mRNA increased 4-fold in C57Bl/6 mice and 2-fold in BALB/c mice. alpha 2I procollagen mRNA was increased only in C57Bl/6 mice (2-fold. TGF-β1 is activated by MMP in both physiological and pathological situations (18). Activated TGF-β1 in turn inhibits MMP-9 and activates TIMP-1,3 (10, 19) to reduce matrix degradation and collagen accumulation. The present study was aimed to evaluate the convergence of the TGF-β1 and protease-antiprotease signaling pathways and their modulation by NAC treatment. The study was designed to evaluate the time course of MMP-9, TIMP-1 and TIMP-3, TGF-β1 expression and collagen deposition in bleomycin-instilled lungs in the presence and absence of NAC treatment. 

## Materials and Methods

***Chemicals and reagents***

In the present study, bleocip (bleomycin sulphate, Cipla Ltd), N-acetyl-cysteine (Mucinac, Cipla Ltd), ketamine hydrochloride (Neon India Ltd), anti-TGF-β1 (SAB 4502954, Sigma Life Sciences, USA), MMP-9 (Sc-8848, SantaCruz Biotechnology, USA), TIMP-1 (Sc-5538, SantaCruz Biotechnology, USA), TIMP-3 (ab155749 Abcam, USA), anti-goat IgG (SAB3700288, Sigma Life Sciences), mouse and rabbit ExtrAvidin^®^ Peroxidase staining Kit (EXTRA2, EXTRA3) Sigma Life Sciences, USA), NovaRED substrate kit (SK-4800 Vector labs, USA), Meyer’s Hematoxylin, Ambion TRIzol^®^ (Invitrogen 15596018), MMLV (M0253S, New England Biolabs), primers (Sigma-Aldrich, India, Table 1), RNase (M0314, New England Biolabs), dNTPs (N0447S, New England Biolabs), random primers (S1330S, New England Biolabs), SyBr Green (S4438 Jumpstart^®^Taq Ready Mix, Sigma Life Sciences), Quantikine TGF-β1 (MB100B, R&D systems), and protease inhibitor cocktail (Sigma Life Sciences) were employed. All chemicals used were of the highest commercial grade available. Bleomycin sulphate was dissolved in sterile 0.9% saline solution and NAC was dissolved in the distilled water. 

***Animals***

Adult male Wistar rats (200-250 g) were obtained from the animal house of V.P. Chest Institute New Delhi. All the animals were maintained in the standard husbandry conditions and were provided with food and water *ad libitum*. The study was approved by the Institute animal ethical committee (VPCI/IAEC/2015/03), and experiments were performed according to National guidelines on the care and use of laboratory animals, India. 

***Rat model of fibrosis***

Bleomycin sulphate was employed to induce lung fibrosis in rats as per reported method (20,21). In brief, animals were anesthetized using ketamine hydrochloride (50 mg/kg IM) and 1% xylocaine was used to induce local anesthesia. Thereafter, the trachea was exposed and 100 µl of bleomycin sulphate (7 units/kg) was instilled inside the trachea using 24G needle. The animals were sacrificed on 7^th^, 14^th ^and 28^th^ day after bleomycin instillation in the trachea.

***Histopathology***

Five μm sections of the formalin fixed, paraffin embedded lung portions were stained with hematoxylin and eosin for histopathological study. The severity of lung inflammation and fibrosis was semi-quantitatively assessed and graded (scale 0 to 8) (22). The major histopathological features examined included the degree of inflammatory cell infiltration, thickening of alveolar interstitium and alveolar architectural changes. 

***Quantification of collagen content in the lungs***

The extent of collagen deposition in the lungs was quantified by assaying the lung hydroxyproline content colorimetrically using p-dimethylbenzaldehyde. The absorbance was measured at 550 nm and values were expressed as mg of collagen/total lung weight (23)sodium hydroxide, p-dimethylaminobenzaldehyde, pH of the reaction buffer, and length of oxidation time were examined to obtain satisfactory results. The method has been applied to samples of tissue homogenate and purified acid soluble collagen, with recovery of added hydroxyproline of 101 +/- 6.5 and 104 +/- 6.0 (SD. 

***Immunohistochemistry***

The lung tissue sections were deparaffinised and antigen retrieval was performed in the microwave using 10 mM sodium citrate buffer, pH 6.0. Endogenous peroxidase quenching and blocking of non-specific antigens was performed using 3% H_2_O_2_ and 4% bovine serum albumin (BSA). The sections were incubated overnight at 4 ^°^C with primary antibodies (1:100) for MMP-9, TGF-β1, TIMP-1 and TIMP-3. The sections were reacted with biotinylated secondary antibody (anti-goat, anti-mouse, anti-rabbit) and extravidin. NovaRED substrate was added. The sections were counterstained with Mayer’s hematoxylin. Immunostaining was quantified using Nikon 90i Eclipse microscope, Japan and NIS-Ar elements image analysis software (24). Briefly, 10 fields (40x) were randomly selected and the intensity of chromogen-positive cells was measured. The maximum intensity of the red/green/blue (RGB) image analyzed was 250. An inverse correlation exists between the pigmentation (amount of antigen) and its numerical value (darker RGB areas have lower intensity values). The reciprocal intensity (RI) was calculated by subtracting the intensity of positively stained cells from 250. The protease-antiprotease balance was calculated as the ratio of mean expression of MMP-9 protein to the TIMP-1,3 by the alveolar epithelial cells (AECs) and interstitial macrophages.

***Quantitative real time PCR (qRT-PCR)***

The total RNA was extracted from the lung tissue homogenates using TRIzol and chloroform. Thereafter, RNA was precipitated with isopropanol and reverse transcribed to cDNA using MMLV-RT enzyme. qRT-PCR was performed on Mastercycler RealPlex 2.0, Eppendorf: PCR activation (95 ˚C for 5 min), denaturation (35 cycles, 95 ^ᵒ^C for 30 sec), annealing (60˚C for 35 sec) and extension (72^ᵒ^C for 30 sec). The relative gene expression (fold change) was calculated using ^ΔΔ^Ct method (25). 

***Quantification of TGF-β1 protein levels in the lungs using ELISA***

The lung tissue was homogenized in the lysis buffer (0.5% TritonX-100, 150 mM NaCl, 15 mM Tris, 1 mM CaCl_2_, 1 mM MgCl_2_ (pH 7.4), and the levels of TGF-β1 protein were estimated using TGF-β1 ELISA kit as per manufacturer’s protocol.

***Experimental protocol***

Seventy two animals were employed in the present study and these were divided into four groups

*Group I (Saline control, n=18)*

In this group, normal saline was instilled in the trachea and animals were sacrificed on 7^th^, 14^th^ and 28^th^ days. The lungs were isolated for histopathological, immunohistochemical, biochemical and ELISA as described above. The time course of mRNA and cellular protein expression of MMP-9, TIMP-1,3, TGF-β1 and hydroxyproline were evaluated in the lung tissues.

*Group II (Bleomycin control, 7 U/kg, n=18)*

In this group, bleomycin sulphate (7 U/kg) was instilled in the trachea and six animals were sacrificed on 7^th^, 14^th^ and 28^th^ days. The lungs were isolated for histopathological, immunohistochemical, biochemical and ELISA as described in group I.

*Group III (NAC, 0.3 mmol/kg in bleomycin control, n=18)*

The treatment with NAC (0.3 mmol/kg/day *PO*) was started 7 days before bleomycin instillation and the animals were sacrificed on days 7, 14 and 28 (n=6 on respective days). The lungs were isolated and analysed as described in group I. 

*Group IV (NAC, 3 mmol/kg in bleomycin control, n=18)*

The treatment with NAC (3 mmol/kg/day *PO*) was started 7 days before bleomycin instillation and the animals were sacrificed on days 7, 14 and 28 (n=6 on respective days). The lungs were isolated and analysed as described in group I. 

***Statistical analysis***

The GraphPad prism 5.0 software was used for statistical analysis. The results were expressed as Means±SEM (Standard error of mean) and analysed using one way ANOVA followed by Newman Keule’s *post hoc* test.* P* value of <0.05 was considered significant.

## Results

***Histopathological alterations in the lungs following bleomycin instillation and NAC treatment***

**Figure 1:**
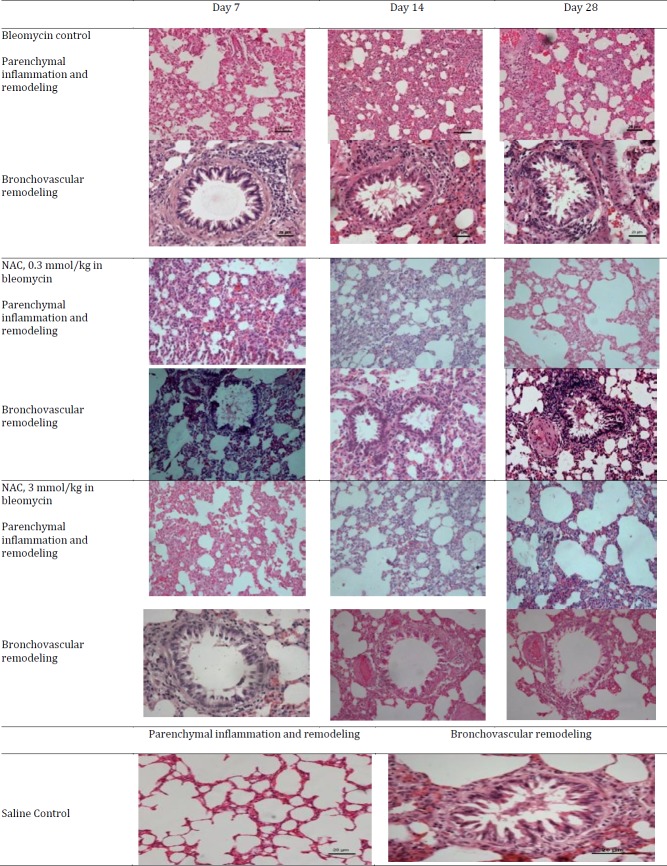
Lung histopathology to assess parenchymal inflammation and remodelling along with bronchovascular remodelling after bleomycin instillation and N-acetylcysteine therapy (hematoxylin and eosin stain). Progressive increase in interstitial, peribronchiolar/perivascular inflammation along with increase in fibrosis was observed on day 7, 14 and 28 in bleomycin instilled rats. Normal lung parenchymal architecture and bronchovascular bundles in saline control groups at all time intervals i.e. 7^th^, 14^th^ and 28^th^ days. Bleomycin-induced parenchymal inflammation and remodelling was significantly reduced on different days in NAC-treated rats

Intratracheal instillation of bleomycin resulted in significant development of histopathological alterations in the lungs on the 7^th^ day assessed in terms of increase in parenchymal, peribronchiolar and perivascular inflammation and remodeling corresponding to grade 1 fibrosis (Ashcroft’s grade, Table 2). This stage corresponded to early cellular phase of injury. A further increase in interstitial inflammation along with the loss of AECs, increase in the number of fibroblasts, and deposition of ECM proteins including collagen corresponding to grade 3 fibrosis was observed on 14^th^ day. This stage corresponded to mid-cellular phase of injury. On day 28, parenchymal and bronchovascular fibrosis corresponding to grade 5 fibrosis was observed along with reduction in inflammation, which represented the fibrotic phase. Treatment with NAC completely reversed bleomycin-induced interstitial inflammation and fibrosis on day 7 (Table 3). It also reduced parenchymal collagen deposition and attenuated interstitial fibrosis to grade 2 on day 14. On day 28, further reduction in parenchymal collagen deposition and decrease in interstitial fibrosis to grade 1 was observed in response to NAC therapy. The effects of different doses of NAC were not significantly different and produced equivalent degree of efficacy in reversing parenchymal fibrosis from day 14 onwards (Figure 1, Table 3). 

***Effect of bleomycin installation and NAC treatment on the hydroxyproline content in the lungs***

**Figure 2:**
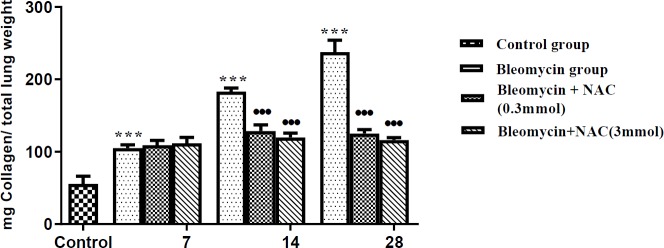
Total lung collagen content in bleomycin instilled animals before and after treatment with N-acetylcysteine. ^***^P<0.001 vs control group; ^•••^*P<*0.001 vs bleomycin group on 14^th^ day and on 28^th^ day

Bleomycin instillation led to significant rise of hydroxyproline levels in the lungs (***P <0.001, Figure 2, ) indicating the rise in the collagen levels on different days i.e. 7^th^, 14^th^ and 28^th^ days in comparison with saline-treated animals. However, treatment with both doses of NAC was effective in significantly reducing the parenchymal collagen on days 14 and 28 (^•••^*P* <0.001, Figure 2).

***Effect of bleomycin instillation and NAC treatment on mRNA and protein expression of MMP-9***

**Figure 3:**
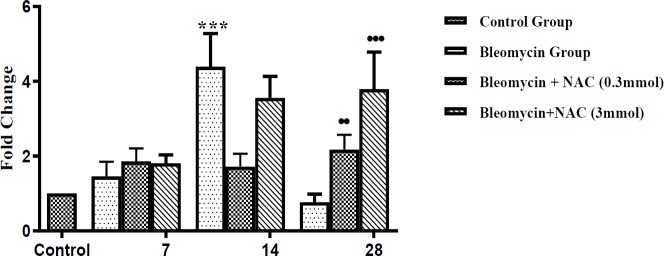
The changes in the mRNA expression of MMP-9 in bleomycin instilled and NAC treated animals. ^***^P<0.001 vs control group; ^••^*P<*0.01 vs bleomycin group on day 28; ^•••^*P<*0.001 vs bleomycin group on day 28 MMP-9: matrix metalloproteinase-9; NAC: N-acetylcysteine

**Figure 4:**
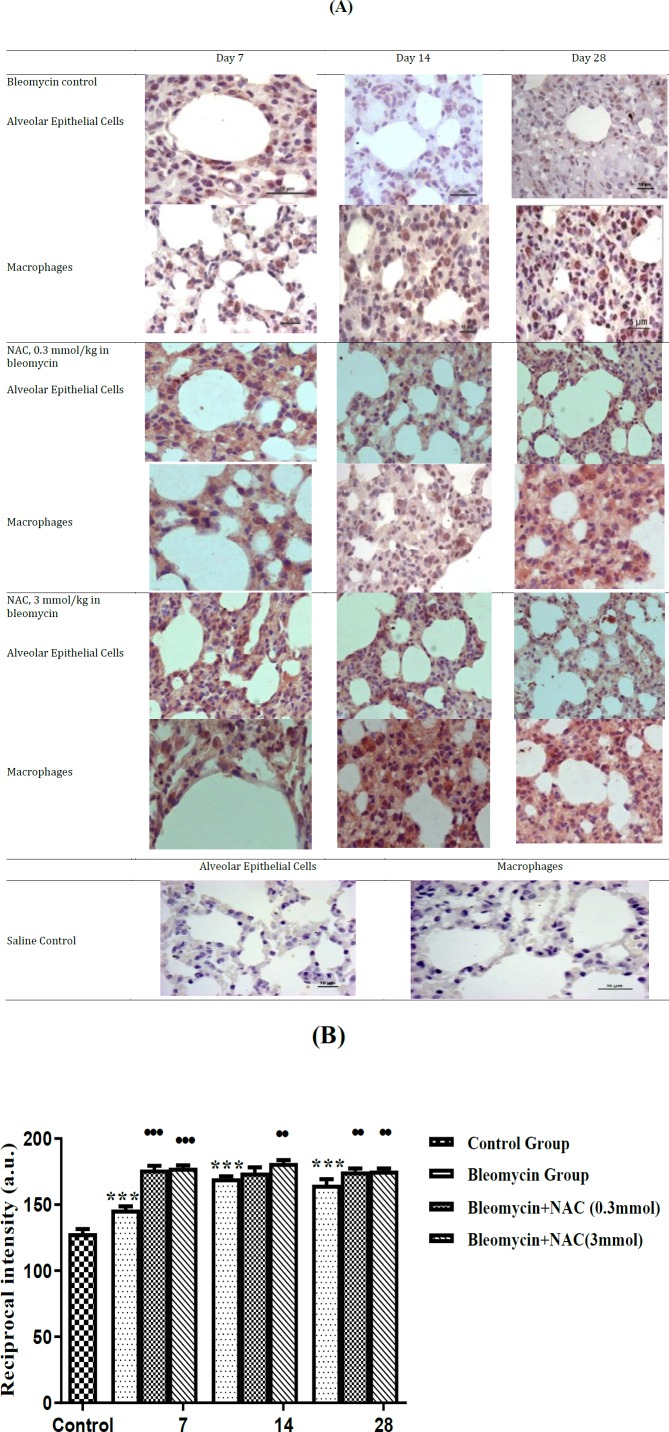
The changes in MMP-9 protein expression in alveolar epithelial cells and macrophages following bleomycin instillation and N-acetylcysteine treatment. MMP-9 is maximally expressed in the inflammatory cells in the interstitium and perivascular region on 14^th^ day. Treatment with NAC attenuated interstitial fibrosis to grade 2 and reduced parenchymal collagen deposition. The MMP-9 protein expression was persistent in the interstitial macrophages, while it was reduced in the perivascular inflammatory cells on 28^th^ day. Treatment with NAC reduced parenchymal collagen deposition The quantification of MMP-9 protein expression in the lung parenchyma of bleomycin instilled and NAC treated animals. ^***^P<0.001 vs control group; ^•••^*P<*0.001 vs bleomycin on day 7; ^••^*P<*0.01 vs bleomycin on day 14 and 28 (4B)

There was an increase in the MMP-9 mRNA expression (about 1.4 fold increase, *P*=ns) on 7^th ^day after bleomycin instillation as compared to normal control (Figure 3). Moreover, there was weak MMP-9 expression in AECs, perivascular inflammatory cells and interstitial macrophages on 7^th^ day (Figure 4A). There was a significant increase in the mRNA (about 4.3 fold,^***^ P<0.001) (Figure 3) and protein expression of MMP-9 (Figure 4B) on 14^th^ day of bleomycin instillation. MMP-9 was strongly expressed in the inflammatory cells of the interstitium and in the perivascular region (Figure 4A). Interestingly, the mRNA (about 1.01 fold increase) and protein expression of MMP-9 gene was normalized on the 28^th^ day. Moreover, the expression of MMP-9 was reduced in AECs, perivascular inflammatory cells and in interstitial macrophages (Figure 4A). The reduction in MMP-9 expression was correlated with the reduction of inflammation on 28^th^ day. Treatment with NAC (3 mmol/kg) significantly increased the mRNA levels of MMP-9 on days 14 and 28. However, NAC treatment (0.3 mmol/kg) significantly increased the mRNA levels of MMP-9 only on day 28 (fold change 2.17, ^•••^* P*<0.001) (Figure 3). There was significant upregulation of protein expression of MMP-9 in bleomycin-instilled animals (Figure 4A). NAC treatment increased the MMP-9 protein expression in AECs and alveolar macrophages on all days of assessment (•••*P*<0.001 vs bleomycin on day 7; ••* P*<0.01 vs bleomycin on days 14 and 28, Figure 4B).

***Effect of bleomycin instillation and NAC treatment on mRNA and protein expression of TIMP-1***

**Figure 5:**
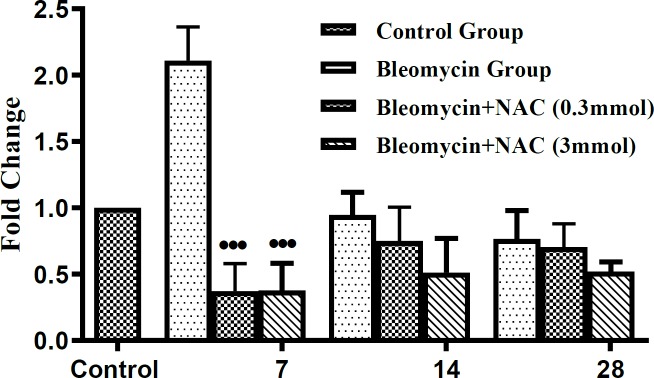
The changes in the Tissue inhibitor of Matrix metalloproteinase-1 (TIMP-1) mRNA expression in bleomycin instilled animals. ^•••^*P<*0.001 vs bleomycin

**Figure 6:**
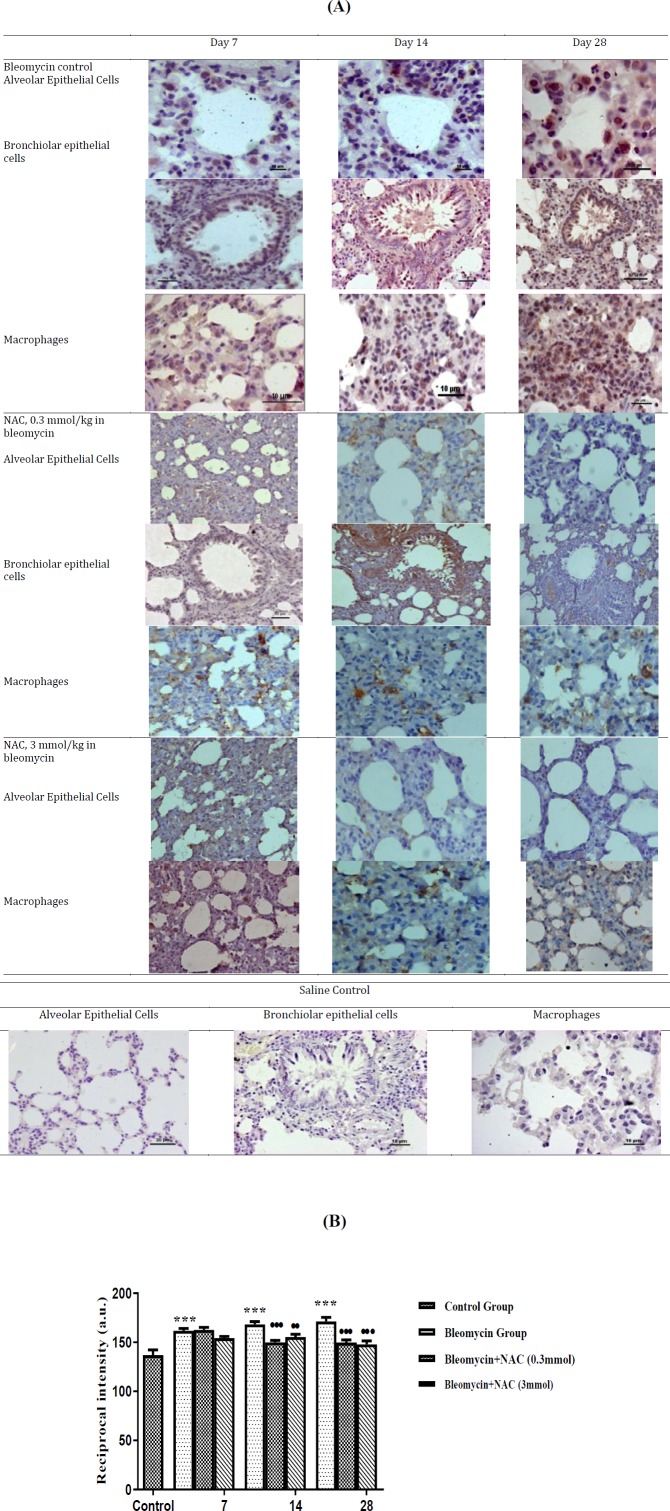
The changes in tissue inhibitor of matrix metalloproteinase-1 (TIMP-1) expression in saline control lungs and after bleomycin instillation in alveolar epithelial cells and bronchial epithelial cells (6A). Quantification of the intensity of TIMP-1 expression in the lung parenchyma. ^***^P<0.001 vs control, ^•••^*P<*0.001 and ^••^*P<*0.01 vs bleomycin (6B)

**Figure 7.**
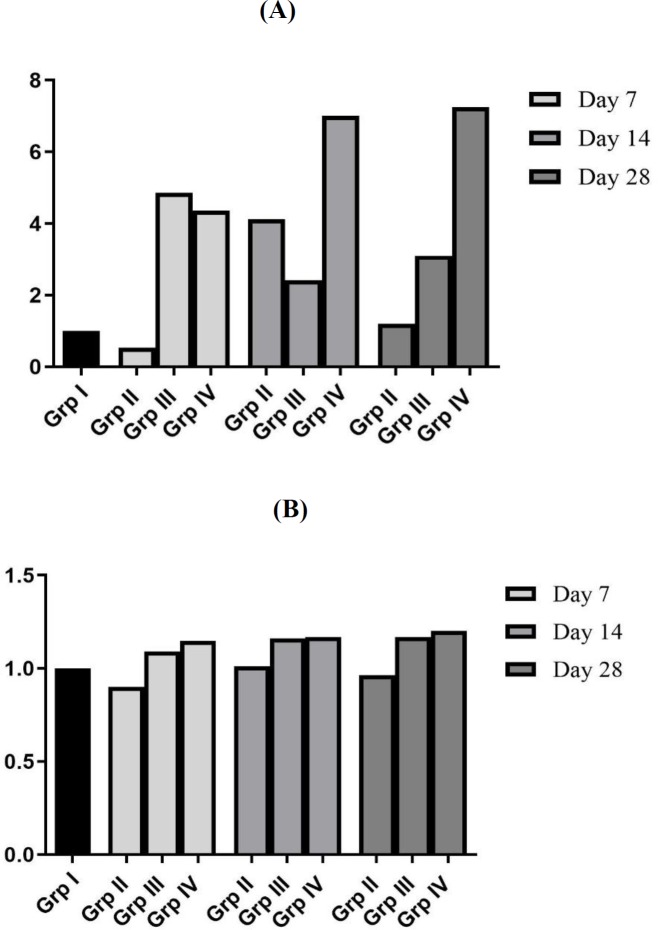
Ratio of mean MMP-9/TIMP-1mRNA (7A) and ratio of mean MMP-9/TIMP-1 protein balance (7B) after bleomycin and NAC treatment in male Wistar rat

Bleomycin instillation led to increase in mRNA (Figure 5; Fold change: 2.62) and protein expression of TIMP-1 in AECs and interstitial macrophages on 7^th^ day (Figure 6A) as compared to normal control group. However, mRNA expression of TIMP-1 returned to baseline level on days 14 and 28 (Fold change of 1.04 and 0.78, respectively) (Figure 5), while TIMP-1 protein expression in AECs and interstitial macrophages remained persistently elevated (Figure 6A). The mRNA ratio of MMP-9/TIMP-1 was approximately ~1 in the control group. After bleomycin instillation, due to rise in the TIMP-1 mRNA levels, there was reversal of MMP-9/TIMP-1 mRNA ratio (<1) on 7^th^ day (Figure 7A). On days 14 and 28, the TIMP-1 mRNA levels returned to baseline with MMP-9/TIMP-1 mRNA ratio of >1. However, the TIMP-1 protein levels were persistently elevated up to 28^th^ day with MMP-9/TIMP-1 protein ratio of ~1 (Figure 7B). Treatment with NAC reduced the TIMP-1 mRNA expression on day 7 (^•••^*P*<0.001, Figure 5), and resulted in significant increase in the MMP-9/TIMP-1 mRNA ratio>1 (day 7 to 28, Figure 7A). NAC treatment also reduced the TIMP-1 protein expression in the AECs and in alveolar macrophages (Figure 6A). It resulted in increase in the MMP-9/TIMP-1 protein ratio>1 (Figure 7B), which shifted the balance between the enzyme and inhibitor and resulted in increase in proteolysis. This correlated with the reduction in the lung parenchymal collagen deposition from day 7 to day 28 (Figure 2).

***Effect of bleomycin instillation and NAC treatment on mRNA and protein expression of TIMP-3***

**Figure 8:**
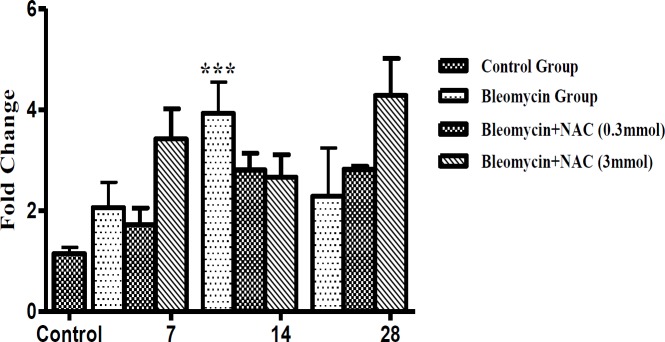
The changes in the tissue inhibitor of matrix metalloproteinase-3 (TIMP-3) mRNA levels in the lung after bleomycin instillation and NAC treatment

^***^P<0.001 vs normal control

**Figure 9:**
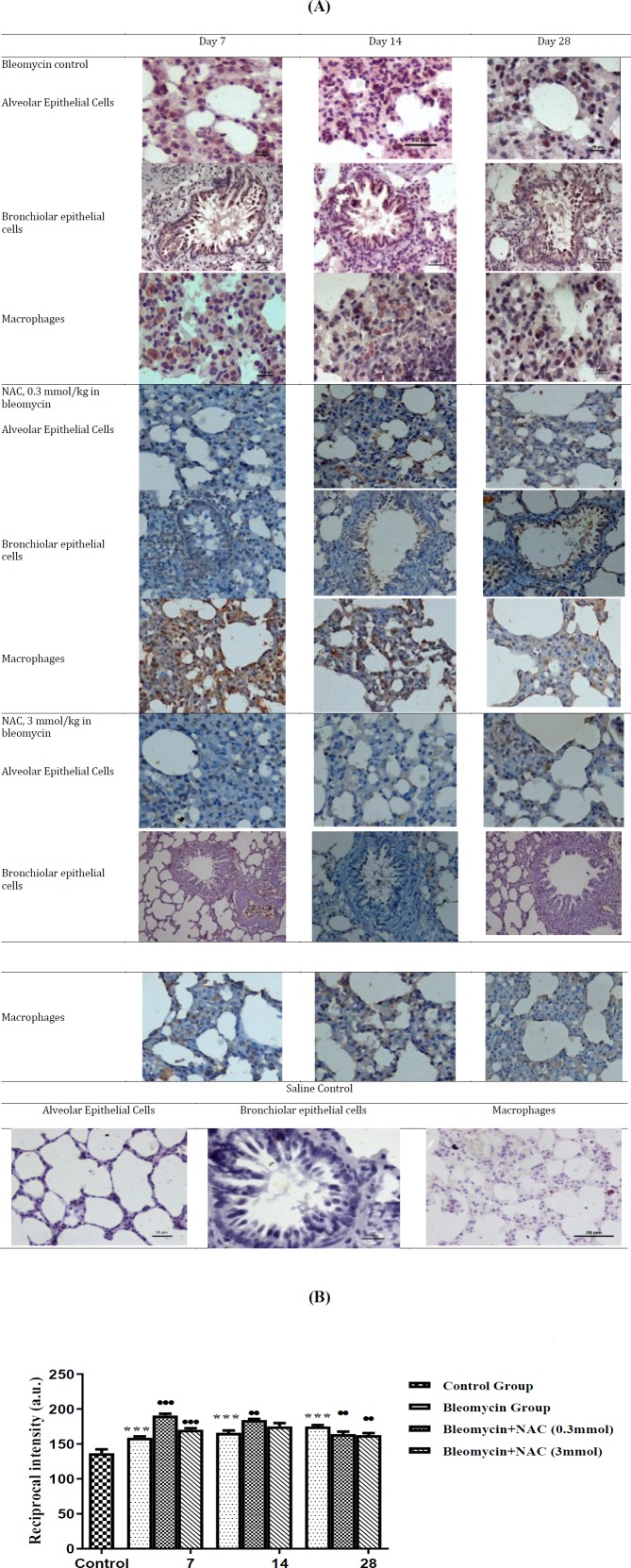
The changes in the TIMP-3 protein expression in saline control lungs and after bleomycin instillation (N=6 in each group) in wistar rat (9A). Quantification of the intensity of TIMP-3 expression in saline control lungs and after bleomycin instillation. ***P<0.001 vs control, ^•••^*P<*0.001, ^••^*P<*0.01 vs bleomycin (9B)

**Figure 10:**
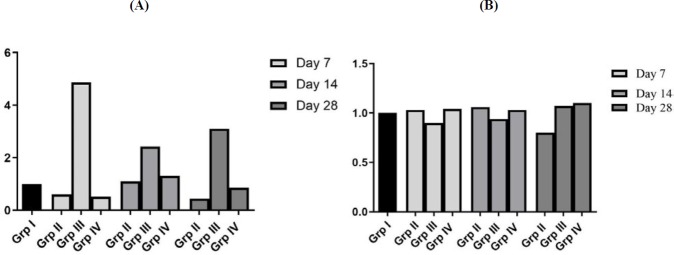
Ratio of mean MMP-9/TIMP-3mRNA (Figure 10A) and ratio of mean MMP-9/TIMP-3 protein balance (10B) after bleomycin and NAC treatment in wistar rat

**Figure 11:**
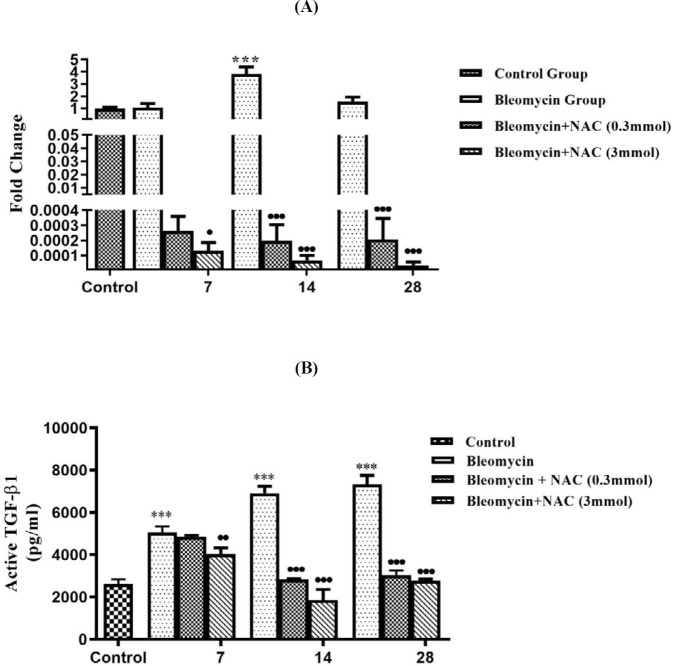
The changes in TGF-β1 mRNA levels ^***^P<0.001 vs normal control in wistar rat; ^•••^*P<*0.001, ^•^*P<*0.05 vs bleomycin. (11A) and active TGF-β1 protein levels after bleomycin and NAC treatment ^••^*P<*0.01 vs bleomycin, ^***^P<0.001 vs normal control, ^•••^*P<*0.001 vs bleomycin (11B)

**Figure 12:**
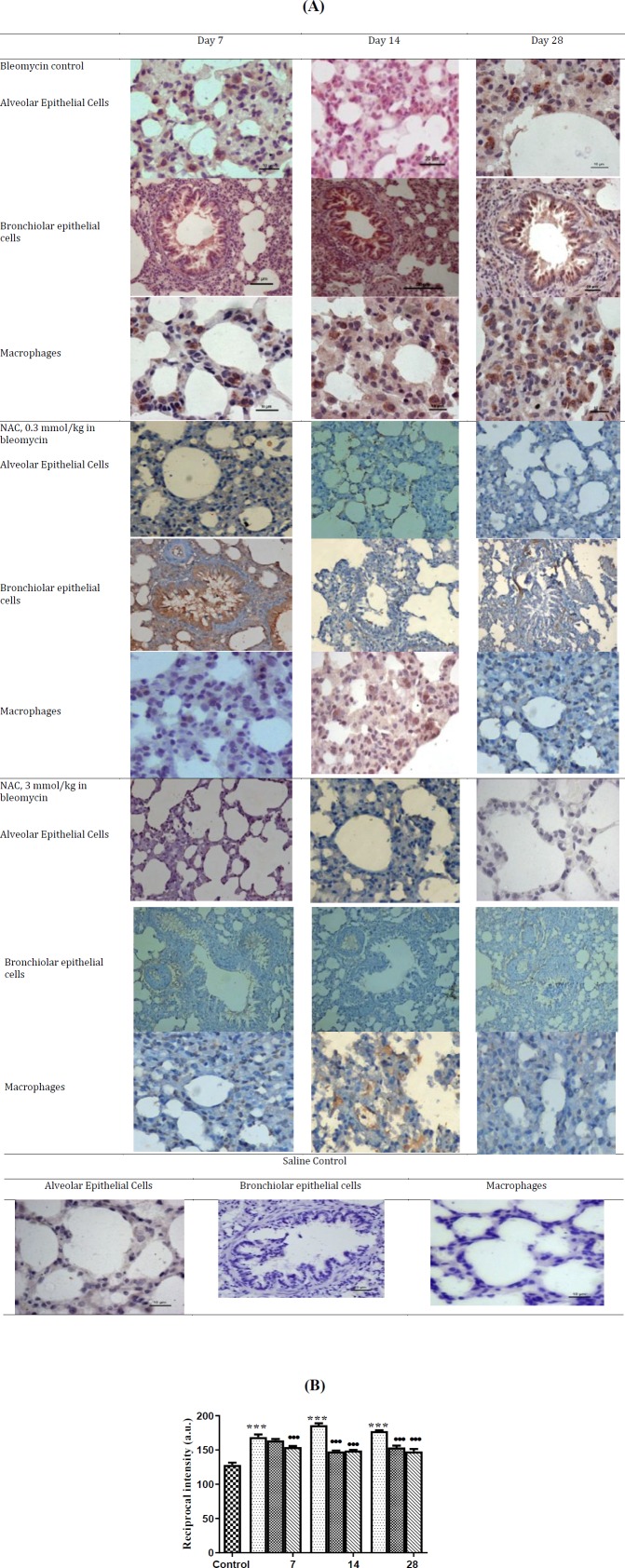
Changes in the transforming Growth factor-β1 (TGF-β1) protein expression in saline control lungs and after bleomycin instillation in wistar rat (12A). Quantification of the intensity of TGF-β1 protein expression in the lung parenchyma. ^***^P<0.001 vs control, ^•••^*P<*0.001 vs bleomycin (12B)

In the present study, a progressive increase in TIMP-3 mRNA and protein expression in AECs, bronchial epithelial cells (BECs) and interstitial macrophages were observed from day 7 onwards up to day 28 after bleomycin instillation (^***^P<0.001, Figure 8 and 9A). Bleomycin injury reversed the protease-antiprotease balance, MMP-9/TIMP-3 mRNA ratio˂1, (Group IV, Figure 10A) and MMP-9/TIMP-3 protein ratio ˂1, Figure 10B. NAC therapy increased the mRNA and protein expression of TIMP-3 (^***^P<0.001, Figure 8, 9B) as well as of MMP-9; therefore, the MMP-9/TIMP-3 balance was unaltered in the presence of NAC (Figures 8, 9B, 10A and 10B). In other words, treatment with NAC increased the expression of both MMP-9 and TIMP-3 to correct bleomycin-induced alteration in MMP-9/TIMP-3 ratio, which eventually prevented aberrant parenchymal remodeling.

was noted as compared to the control group (Figure 11A, 11B). On day 14, the lung TGF-β1 mRNA significantly increased and returned to baseline on day 28 (***P<0.001, Figure 11A). Progressive increase in the TGF-β1 protein expression was noted in AECs, BECs, and interstitial macrophages on days 14 and 28 (^***^P<0.001, Figure 12A and 12B). Treatment with NAC significantly reduced the TGF-β1 mRNA expression at all time intervals (Figure 11A), which was correlated with reduction in cellular protein expression from day 7 onwards up to day 28 (^•••^*P*<0.001 Figure 11B, 12A and 12B).

**Table 1:** Forward and Reverse primer sequences for PCR analysis

**Gene**	**Forward Primer (5’-3’)**	**Reverse Primer (5’-3’)**
TGF-β1(133bp)	TTTGGAGCCTGGACACACAG	TTGCGACCCACGTAGTAGAC
MMP-9(105bp)	CTCGGATGGTTATCGCTGGT	AGTTGCCCCCAGTTACAGTG
TIMP-1(156bp)	TTTCCCTGTTCAGCCATCCC	ACCCCAAGGTATTGCCAGG
TIMP-3(155bp)	GCCTCAATTACCGCTACCA	ATGCAGGCGTAGTGTTTGGA
β-actin (110bp)	GACCTTAACACCCCAGCCA	GTCACGCACGATTTCCCTCTC

**Table 2:** Semiquantitative grading of parenchymal inflammation, vascular smooth muscle cell hypertrophy (VSMCH) and Ashcroft’s grade of fibrosis (†) after bleomycin instillation

Semiquantitative grading of Histopathological changes	Group I Control	Group II (Bleomycin)			
		Day 0	Day 7	Day 14	Day 28
Interstitial inflammation	-	-/+	++	+++	+
Peribronchiolar/Perivascular inflammation	-	+/-	++	+++	+
Interstitial fibrosis	-	-	†	†††	†††††
VSMCH	-	-	+	++	++
Vasoconstriction	-	-	-/+	+	+

VSMCH: Vascular smooth muscle cell hypertrophy

**Table 3:** Effect of N-acetylcysteine (NAC) on bleomycin induced lung parenchymal and vascular histopathological changes

Effect of NAC	Group III Day 7		Group III Day 14		Group III Day 28	
Sub-group	IIIA	IIIB	IIIA	IIIB	IIIA	IIIB
Interstitial inflammation	-/+	-	+	-/+	-	-
Peribronchiolar/ Perivascular inflammation	-/+	-	+	-/+	-	-
Interstitial fibrosis	-/†	-	††	††	†	†
VSMCH	+	+	++	++	++	++
Vasoconstriction	-/+	-/+	+	+	+	+

+: Grade of inflammation and Vascular smooth muscle cell hypertrophy (VSMCH)

†: Ashcrofts grade of fibrosis

## Discussion

In the present study, bleomycin instillation in the trachea resulted in significant histopathological changes in the lungs of the rats assessed in terms of progressive increase in parenchymal, peribronchiolar and perivascular inflammation along with fibrosis from day 7 to day 28. Moreover, there was an increase in the collagen content in the lung parenchyma again suggesting the development of fibrosis in bleomycin-exposed animals. Indeed, the development of pneumonitis (26) and pulmonary fibrosis (27) are major causes of pulmonary toxicity in patients with cancer undergoing chemotherapy with bleomycin. Experimentally, bleomycin-induced fibrosis in rodents has been a well-documented model of pulmonary fibrosis (28). In the present study, administration of NAC in bleomycin-instilled animals led to significant improvement in the architecture of lungs and bleomycin-induced histopathological changes, i.e. inflammation and fibrosis, were significantly attenuated. These beneficial effects of NAC in bleomycin-induced pulmonary observed in this study are consistent with the previous studies documenting the decrease in fibrosis and collagen deposition in lungs during bleomycin exposure (3, 4). 

In order to understand the mechanisms involved in imparting beneficial mesenchymal effects of NAC in bleomycin-induced fibrosis, the changes in the expression of proteolytic and anti-proteolytic enzymes were assessed. In the present study, bleomycin instillation increased the MMP-9 protein expression in the inflammatory cells and AECs from days 7 to 14, which was returned to normal on the day 28. Bleomycin instillation also increased the mRNA and protein expression of TIMP-1 in AECs and interstitial macrophages on 7^th^ day. The TIMP-1 protein expression remained elevated up to 28^th^ day, while expression of MMP-9 decreased, resulting in MMP-9/TIMP-1 protein ratio of less than 1. In other words, bleomycin-induced pulmonary fibrosis on 28^th^ day was associated with a significant decrease in MMP-9/TIMP-1 ratio, signifying decrease in MMP-9 activity and increase in TIMP-1 activity. MMP-9 is a proteolytic enzyme and its overexpression in macrophages has been shown to attenuate pulmonary fibrosis induced by bleomycin (29). TIMP-1 is an inhibitor of MMP, which inhibits the activation of MMP9 from pro-MMP9 by forming a pro-MMP9-TIMP1 complex (30, 31). An increase in the TIMP expression is also associated with decreased collagenase activity (32). Thus, the higher expression of TIMPs inhibits the degradation of fibrillar collagen leading to development of lung fibrosis (33). Thus, a ratio of MMP-9/TIMP-1 becomes critical in determining the development of fibrosis or degradation of collagen to inhibit fibrosis. Earlier studies have shown that the reduction in MMP-9/TIMP-1 ratio is important in promoting bleomycin-induced pulmonary fibrosis (34), which is well-supported by the results of present study showing decrease in MMP-9/TIMP-1 ratio during bleomycin-induced pulmonary fibrosis. 

Similar to the effects on the TIMP-1, NAC treatment also increased the expression of another inhibitor of MMP i.e. TIMP-3 in the present study. TIMP-3 is a potent inhibitor of MMPs (35) and is crucial for the resolution of chronic lung inflammation and injury (36). TIMP-1 is a secreted protein, while TIMP-3 is a membrane bound protein and is restricted to the ECM (37). Bleomycin instillation led to decrease in MMP-9/TIMP-3 ratio, and NAC treatment reversed the effects of bleomycin by normalizing the ratio of MMP-9/TIMP-3 to 1. Accordingly, it is proposed that NAC may inhibit bleomycin-induced development of pulmonary fibrosis by increasing the MMP-9/TIMP-1 and MMP-9/TIMP-3 ratios, which eventually increases the degradation of collagen in the lungs to prevent its excessive deposition in the form of fibrosis. 

Moreover in the present study, there was a marked increase in the mRNA expression of TGF-β and intense immunostaining of reactive AECs, BECs, alveolar macrophages and ECM following belomycin instillation. The increased activation and function of the TGF-β1 complex may possibly occur via MMP-dependent mechanism (38). In present study, a significant increase in MMP-9 mRNA levels was associated with increase in TGF-β1 mRNA expression suggesting the role of MMP-9 in activating the TGF-β1. The activated form of TGF-β1 in turn inhibits the expression of MMP-9 and stimulates the TIMP-1 mRNA expression, shifting the parenchymal remodeling from cellular to fibrotic phase (19). Moreover, TGF-β1 is shown to upregulate the TIMP-3 levels in the primary fibroblast cultures from IPF patients (10). These findings were also observed in this present study, where an increase in the TGF-β1 levels were associated with the concurrent upregulation of TIMP-1 and 3 levels and shifting the ratio of MMP-9/TIMP to <1. Therefore, it may be suggested that TGF-β1 may play an important role in altering the MMP-9/TIMP balance towards TIMPs, thereby creating a microenvironment that favors ECM deposition. Moreover in the present study, treatment with NAC suppressed the production of TGF-β1 and increased the MMP-9/TIMP ratio favoring the degradation of collagen and inhibition of fibrosis. An earlier *in vitro* study has shown that NAC suppresses the production of TGF-β1 by alveolar macrophages in IPF (39). To best of our knowledge, it is the first study documenting that NAC treatment ameliorates bleomycin-induced pulmonary fibrosis by increasing the MMP-9/TIMP ratio and decreasing the production of TGF-β1 in rats. 

## Conclusion

 NAC may directly attenuate mesenchymal remodeling by normalizing the protease-antiprotease balance and favoring the degradation of collegen to reduce fibrosis. In addition to antioxidant effects, NAC-induced lowering of TGF-β1 may also contribute significantly in attenuating bleomycin-induced pulmonary fibrosis, especially when initiated in early cellular phase of disease manifestation. 
